# Trial of labor after caesarean section in low risk pregnancies: is it risky?

**DOI:** 10.1007/s00404-024-07700-1

**Published:** 2024-08-29

**Authors:** Sven Kehl, Hanna Düster, Christel Weiss, Simon Bader, Michael Schneider, Matthias W. Beckmann, Ulf Dammer, Jutta Pretscher

**Affiliations:** 1 Department of Obstetrics and Gynecology, Erlangen University Hospital, Universitätsstr. 21-23, 91054 Erlangen, Germany; 2 Department of Medical Statistics and Biomathematics, University Medical Center Mannheim, Heidelberg University, Mannheim, Heidelberg, Germany; 3 Department of Obstetrics and Gynaecology, St. Theresien Hospital, Nuremberg, Germany

**Keywords:** TOLAC, VBAC, Uterine rupture, Prior caesarean section, Caesarean section rate, Composite adverse outcome

## Abstract

**Purpose:**

To evaluate the influence of a previous caesarean section on adverse composite maternal and perinatal outcome in women who attempted a trial of labor.

**Methods:**

This historical cohort study analyzed maternal and perinatal outcome in women with otherwise low risk pregnancies at term who underwent a trial of labor after a caesarean section (TOLAC). The primary outcome measure was the adverse composite outcome. Secondary outcome measures were amongst others the caesarean section rate and the mode of vaginal delivery.

**Results:**

The adverse composite outcome was more frequently in the previous caesarean section group compared to women with no previous caesarean Sect. (22.3% vs. 15.6%, p < 0.0001). The percentage of caesarean Sect. (15.4% vs. 8.2%, p < 0,0001), uterine rupture (1.0% vs. 0.02%, p < 0.0001), placental abruption (1.1% vs. 0.3%, p = 0.0014), vaginal operative delivery (16.0% vs. 8.6%, p < 0.0001), pH < 7.10 (3.7% vs. 2.5%, p = 0.0151), base excess < -12 (3.2% vs. 2.2%, p = 0.0297), abnormal cardiotocography (22.5% vs. 13.9%, p < 0,0001) and fetal blood analysis (6.2% vs. 2.6%, p < 0.0001) was significantly higher in women with a previous caesarean section. Taking the parity into account, these differences could only been seen in women without a previous vaginal delivery. In parous women with a previous vaginal delivery and a caesarean section in history, the adverse composite did not differ between the groups. Only the rate of pH < 7.1 was higher in women after a caesarean Sect. (4.5% vs. 1.8%, p = 0.0436).

**Conclusion:**

Trial of labor after caesarean in otherwise low risk pregnancies is associated with a higher rate of complications especially if there is no history of vaginal delivery.

## What does this study add to the clinical work

**Table:** 

This study shows that TOLAC carries higher risks, even in a low-risk collective, and highlights the importance of conducting it in settings with immediate access to pediatric care and emergency cesarean section. A key strength is the large sample size and thorough evaluation of TOLAC risk.	

## Introduction

Worldwide, the caesarean section rate still continues to increase [1, 2]. In 2021, worldwide 21.1% of all live births were delivered via caesarean section [2] and even 30.9% in Germany [3]. One reason for the rising caesarean section rate is the high amount of women who deliver via repeat caesarean section. One-third of all caesarean sections in Germany in 2021 were performed because of a previous one [4, 5]. Trial of labor after caesarean (TOLAC) seems to be a safe alternative to elective repeated caesarean section (ERCS) for many women [6–12]. The success rate for vaginal delivery after caesarean section ranges between 60 and 80% [13–15].

In Germany, the birth setting in these cases is a matter of debate. TOLAC at home or in birth centers was an option for pregnant women according to a bilateral agreement between the head organization of health insurances and midwives [16]. According to current national and international guidelines, however, a continuous cardiotocography (CTG) in TOLAC is mandatory [6, 8, 10, 11]. One of the major concerns is the risk of uterine rupture [13, 17]. An abnormal CTG is the most common sign being suggestive for an uterine rupture, and it is reported to be associated with this emergency in up to 70 percent [10]. Therefore, the continuous monitoring of fetal heart rate by CTG was rated obligatory to allow immediate emergency caesarean section for women attempting a vaginal birth after caesarean (VBAC) [6, 8, 10, 11].

But these recommendations are not universally accepted as continuous CTG during labor has also been reported to be associated with a higher rate of caesarean section [18] and the risk of uterine rupture with TOLAC is considered low (between 0.2 and 0.7%) [7, 8, 19–21]. Although the benefits and risks of ERCS and VBAC are well studied, data concerning the influence of a previous caesarean section on maternal and perinatal outcome in otherwise low-risk pregnancies are scarce.

The aim of this study was therefore to evaluate the maternal and perinatal outcome in women with otherwise low-risk pregnancies at term who underwent a trial of labor after caesarean section.

## Methods

This was a historical cohort study in a single tertiary referral center between January 2013 and December 2022. Women with singleton pregnancies in cephalic presentation ≥ 37 + 0 weeks of pregnancy were included. Exclusion criteria were hypertensive disorders of pregnancy, gestational diabetes, intrahepatic cholestasis of pregnancy, fetuses with abnormalities in CTG or fetal doppler parameters, abnormal amniotic fluid (oligohydramnios or polyhydramnios), fetal growth restriction, abnormal fetal size (small or large for gestational age), placenta praevia, intrauterine fetal death and structural or chromosomal fetal malformation. Cases with induction of labor, ERCS and history of more than one caesarean section were also excluded.

Data were collected in daily routine and were pseudonymized. Therefore, an informed consent was not sought for its retrospective analysis. Though, the trial was approved by the local Ethics committee of Friedrich-Alexander-University Erlangen (No. 23-480-Br). According to current recommendations, gestational age was determined by women’s first day of last menstrual period and adjusted with sonographic data of crown-rump-length in the first trimester when gestational age was discrepant more than seven days [22].

Primary outcome parameter was the adverse composite outcome that included the following parameters: neonatal death, maternal death, caesarean section rate, arterial umbilical cord pH < 7, base excess ≤ −16, Apgar score after 5 min < 5, transfer to neonatal care unit, uterine rupture, and placental abruption. Secondary outcome parameters were amongst others the mode of vaginal delivery, meconium stained amniotic liquor, abnormal CTG, fetal blood analysis, and suspected triple I. Triple I was defined as suspected inflammation, infection or both, with maternal fever > 39 °C and one of the following criteria: fetal tachycardia > 160 beats/minute > 10 min or maternal white blood cell count > 15,000/µl without applied corticosteroids.

### Statistical analyses

Qualitative factors are presented by absolute frequencies and percentage values. For quantitative variables, mean values and standard deviations have been calculated. For the comparison of two mean values 2 sample t test has been used for data approximately normally distributed. For ordinally scaled variables (i.e. Apgar score) Wilcoxon two sample test has been performed. The comparison between two groups regarding a qualitative factor has been done with the Chi2 test. If the preconditions of the Chi2 test have not been met, Fisher’s exact test has been used instead. In general, a test result with p less than 0.05 has been considered as statistically significant. All statistical calculations were done with the SAS software, release 9.4 (SAS Institute Inc., Cary, North Carolina, USA).

## Results

In total, there were 25,196 deliveries in the study period. After monitoring of in- and exclusion criteria, 10,808 women could be included in the trial. There were 9812 deliveries without a caesarean section in history and 996 women with a previous caesarean section.

The demographic parameters are given in Table 1. In the previous caesarean section group, more women had no previous vaginal delivery (86.3% vs. 64.8%, p < 0.0001), were older (33.3 ± 4.1 years vs. 31.2 ± 4.7 years, p < 0.0001), had a higher body mass index (BMI in kg/m^2^) (28.1 ± 4.6 vs. 27.9 ± 4.9, p = 0.0023), and the fetal weight was higher (3016.5 ± 1235.0 g vs. 2798.0 ± 1377.2 g, p < 0.0001) compared to the no previous caesarean section group.

**Table 1:** Demographic parameters for women without induction of labor

	No previous caesarean section group (n = 9812)	Previous caesarean section group (n = 996)	p-value
Maternal age (years)	31.2 ± 4.7	33.3 ± 4.1	< 0.0001
No previous vaginal delivery (n, %)	6354 (64.8%)	860 (86.3%)	< 0.0001
BMI (kg/m2)	27.6 ± 4.9	28.1 ± 4.6	0.0023
Gestational age (days)	278.4 ± 6.9	278.2 ± 6.8	0.4523
Fetal weight (grams)	2798.0 ± 1377.2	3016.5 ± 1235.0	< 0.0001
PROM (n, %)	2844 (29.0%)	279 (28.0%)	0.5187

The outcome parameters are given in Table 2. The adverse composite outcome was significantly different between the two groups (22.3% vs. 15.6%, p < 0.0001). Caesarean Section (15.4% vs. 8.2%, p < 0.0001), uterine rupture (1.0% vs. 0.02%, p < 0.0001), and placental abruption (1.1% vs. 0.3%, p = 0.0014) were more common in women with previous caesarean section. The percentage of vaginal operative delivery (16.0% vs. 8.6%, p < 0.0001), pH < 7.10 (3.7% vs. 2.5%, p = 0.0151), base excess < −12 (3.2% vs. 2.2%, p = 0.0297), abnormal cardiotocography (22.5% vs. 13.9%, p < 0.0001) and fetal blood analysis (6.2% vs. 2.6%, p < 0.0001) was significantly higher too for women with previous section.

**Table 2:** Outcome measures in cases with spontaneous onset of labor

	No previous caesarean section group (n = 9812)	Previous caesarean section group (n = 996)	p-value
Adverse composite outcome (n, %)	1535 (15.6%)	222 (22.3%)	< 0.0001
Neonatal death (n, %)	0	1 (0.1%)	0.0922
Maternal death (n, %)	0	0	n.c
Caesarean section (n, %)	807 (8.2%)	153 (15.4%)	< 0.0001
pH < 7.00	25 (0.3%)	5 (0.5%)	0.1913
Base excess < −16	31 (0.3%)	6 (0.6%)	0.1484
Apgar score after 5 min < 5 (n, %)	29 (0.3%)	5 (0.5%)	0.2371
Transfer to neonatal care unit (n, %)	826 (8.4%)	90 (9.0%)	0.5047
Uterine rupture (n, %)	2 (0.02%)	10 (1.0%)	< 0.0001
Placental abruption (n, %)	32 (0.3%)	11 (1.1%)	0.0014
Mode of vaginal delivery			
Spontaneous delivery	8227 (91.4%)	708 (84.0%)	< 0.0001
Vaginal operative delivery	778 (8.6%)	135 (16.0%)	
pH < 7.10	240 (2.5%)	37 (3.7%)	0.0151
Base excess < −12	210 (2.2%)	32 (3.2%)	0.0297
Apgar score after 5 min	9.7 ± 0.8	9.6 ± 0.9	0.0020
Meconium stained liquor (n, %)	1282 (13.1%)	146 (14.7%)	0.1572
Abnormal cardiotocography (n, %)	1368 (13.9%)	224 (22.5%)	< 0.0001
Fetal blood analysis (n, %)	259 (2.6%)	62 (6.2%)	< 0.0001
Suspected triple I (n, %)	27 (0.3%)	4 (0.4%)	0.5255
Shoulder dystocia (n, %)	76 (0.8%)	10 (1.0%)	0.4374

The most frequent indication for a caesarean section were arrest of labor, abnormal CTG, and abnormal fetal blood analysis (Table 3). There were no significant differences between the two groups but the indication for suspected uterine rupture. In women with previous caesarean section, this indication was more common (3.9% vs. 0%, p = 0.0007).

**Table 3:** Indication for caesarean section in cases with spontaneous onset of labor

	No previous caesarean section group (n = 408)*	Previous caesarean section group (n = 127)*	p-value global	p values comparisons (yes/no)
Abnormal cardiotocography (n, %)	130 (31.9%)	45 (35.4%)	0.0053	0.4539
Abnormal fetal blood analysis (n, %)	14 (3.4%)	8 (6.3%)		0.1552
Arrest of labor (n, %)	233 (57.1%)	64 (50.4%)		0.1836
Suspected placental abruption (n, %)	7 (1.7%)	1 (0.8%)		0.6872
Suspected uterine rupture (n, %)	0	5 (3.9%)		0.0007
Suspected triple I (n, %)	14 (3.4%)	2 (1.6%)		0.3808
Others (n, %)	10 (2.5%)	2 (1.6%)		0.7402

*Numbers of caesarean sections: 807 and 153

Taking into account whether a vaginal birth has taken place beforehand, a different picture emerges. In Table 4, the outcome measures in women without a previous vaginal delivery are given. These results are not different from the reported ones. However, when analyzing the outcome parameters of parous women with a previous vaginal delivery, the adverse composite outcome was not significantly different (11.8% vs. 7.7%, p = 0.0814, Table 5). The only parameter that was different between the two groups in women with previous vaginal delivery was the rate of umbilical cord pH < 7.10 (4.5% vs. 1.8%, p = 0.0436).

**Table 4:** Outcome measures in women without previous vaginal delivery and spontaneous onset of labor

	No previous caesarean section group (n = 6354)	Previous caesarean section group (n = 860)	p-value
Adverse composite outcome (n, %)	1269 (20.0%)	206 (23.6%)	0.0130
Neonatal death (n, %)	0	1 (0.12%)	0.1191
Maternal death (n, %)	0	0	nc
Caesarean section (n, %)	737 (11.6%)	147 (17.1%)	< 0.0001
pH < 7.00	16 (0.25%)	5 (0.59%)	0.0953
Base excess < −16	24 (0.4%)	6 (0.7%)	0.1618
Apgar score after 5 min < 5 (n, %)	20 (0.32%)	5 (0.58%)	0.2113
Transfer to neonatal care unit (n, %)	633 (10.0%)	80 (9.3%)	0.5428
Uterine rupture (n, %)	2 (0.03%)	10 (1.2%)	< 0.0001
Placental abruption (n, %)	19 (0.3%)	9 (1.1%)	0.0039
Mode of vaginal delivery			
Spontaneous delivery	4900 (87.2%)	580 (81.4%)	< 0.0001
Vaginal operative delivery	717 (12.8%)	133 (18.7%)	
pH < 7.10	177 (2.8%)	31 (3.6%)	0.1745
Base excess < −12	166 (2.6%)	30 (3.5%)	0.1395
Apgar score after 5 min	9.6 ± 0.8	9.6 ± 0.9	0.0186
Meconium stained liquor (n, %)	858 (13.5%)	128 (14.9%)	0.2687
Abnormal cardiotocography (n, %)	1079 (17.0%)	213 (24.8%)	< 0.0001
Fetal blood analysis (n, %)	222 (3.5%)	58 (6.7%)	< 0.0001
Suspected triple I (n, %)	23 (0.36%)	4 (0.5%)	0.5552
Shoulder dystocia (n, %)	34 (0.54%)	7 (0.8%)	0.3283

*nc* not calculable

**Table 5:** Outcome measures in parous women (with previous vaginal delivery) and spontaneous onset of labor (no induction of labor)

	No previous caesarean section group (n = 3454)	Previous caesarean section group (n = 134)	p-value
Adverse composite outcome (n, %)	265 (7.7%)	16 (11.8%)	0.0814
Neonatal death (n, %)	0	0	nc
Maternal death (n, %)	0	0	nc
Caesarean section (n, %)	70 (2.0%)	6 (4.4%)	0.0663
pH < 7.00	9 (0.3%)	0	1.0000
Base excess < −16	7 (0.20%)	0	1.0000
Apgar score after 5 min < 5 (n, %)	9 (0.3%)	0	1.0000
Transfer to neonatal care unit (n, %)	192 (5.6%)	10 (7.5%)	0.3731
Uterine rupture (n, %)	0	0	nc
Placental abruption (n, %)	13 (0.4%)	2 (1.5%)	0.1085
Mode of vaginal delivery			
Spontaneous delivery	3294 (98.2%)	128 (98.5%)	1.0000
Vaginal operative delivery	60 (1.8%)	2 (1.5%)	
pH < 7.10	63 (1.8%)	6 (4.5%)	0.0436
Base excess < −12	43 (1.3%)	2 (1.5%)	0.6929
Apgar score after 5 min	9.8 ± 0.7	9.8 ± 0.4	0.5731
Meconium stained liquor (n, %)	422 (12.2%)	18 (12.2%)	0.7226
Abnormal cardiotocography (n, %)	289 (8.4%)	11 (8.1%)	0.9082
Fetal blood analysis (n, %)	37 (1.1%)	4 (2.9%)	0.06677
Suspected triple I (n, %)	4 (0.1%)	0	1.0000
Shoulder dystocia (n, %)	42 (1.2%)	3 (2.2%)	0.2419

*nc* not calculable

## Discussion

The present study evaluated the peripartum and perinatal outcome in women who underwent a TOLAC in otherwise low-risk pregnancies. This question is of great clinical interest and the subject of current controversial discussions. The analysis of the data showed that TOLAC is associated with a higher risk, even if no other risk factors (low-risk pregnancy) are present. This risk is particularly high in women who have never had a vaginal birth before.

The adverse composite outcome was made up of various individual parameters. Perinatal mortality was not different in this cohort, whereas other investigators published recently an increased perinatal mortality in TOLAC [23, 24]. The individual parameters that were significantly different between the two groups were the cesarean section rate (p < 0.0001), the rate of uterine rupture (p < 0.0001) and the rate of placental abruption (p = 0.0014). The caesarean section rate (15.4% vs. 8.2%, p < 0.0001) and the vaginal operative delivery rate were higher in the previous caesarean section group (16.0% vs. 8.6%, p < 0.0001). In the subgroup of women without a previous vaginal delivery, the caesarean section rate and the vaginal operative delivery rate were higher in the previous caesarean section group (17.1% vs. 11.6%, p < 0.0001, and 18.7% vs. 12.8%, p < 0.0001), too. In the subgroup of women with a previous vaginal delivery, no difference concerning the caesarean section rate and the vaginal operative delivery rate could be demonstrated. These increased rates are similar to the ones reported by Vaajala et al. The rate of vaginal operative delivery in the TOLAC group was also significantly higher (16% versus 3%, p < 0.001) in this study [23]. The vaginal delivery rate after previous caesarean section differs between countries. In the present study, the rate of spontaneous vaginal delivery was 84% in women with previous caesarean section and 91% in women without previous caesarean section. In the US, the VBAC rate is reported to range between 39 and 70% [25], whereas this rate is reported to be 67% in Finland [23] and 85% in Taiwan [26].

Uterine rupture was more frequent in the previous caesarean section group, not only in the total group (1.0% vs. 0.02%, p < 0.0001), but also in women without a previous vaginal delivery (1.2% vs. 0.03%, p < 0.0001). In parous women with a previous vaginal delivery, there was no uterine rupture in both groups. The percentage of uterine rupture corresponds to the values previously reported in other publications. The ACOG guideline reported a 0.7% risk of uterine rupture in women with a TOLAC [10]. In other countries, the reported rates are lower, e.g. 0.5% in Austria, Australia and Canada [7, 8, 19], and 0.2% in Scotland [20].

The distribution of placental abruption was similar. The rates of placental abruption was significantly different in the total group (1.1% vs. 0.3%, p = 0.0014) as well as in the group of women without a previous vaginal birth (1.1% vs. 0.3%, p = 0.0039). It is known that a previous caesarean section is associated with higher rates of placental abruption [27].

The rates of abnormal CTG and fetal blood analysis were also significantly higher in the overall population (22.5% vs. 13.9%, p < 0.0001, and 6.2% vs. 2.6%, p < 0.0001) and in women without a history of vaginal delivery (24.8% vs. 17.0%, p < 0.0001, and 6.7% vs. 3.5%, p < 0.0001). They did not differ in the subgroup of women with a previous vaginal birth (p = 0.9082, and p = 0.06677). There is little data on the frequency of abnormal CTG and fetal blood analysis in women with TOLAC.

No difference was found in terms of postnatal outcome. Apgar score < 5 after 5 min (p = 0.2371), pH < 7.00 (p = 0.1913) and base excess < −16 (p = 0.1484) were not different between the groups. There was also no difference in the rate of transfer to the neonatal care unit (p = 0.5047). In the total study group, umbilical cord pH < 7.10 (3.7% vs. 2.5%, p = 0.0151) and base excess < −12 (3.2% vs. 2.2%, p = 0.0297) were found more often in women with a history of cesarean section. When the subgroups “women with a previous vaginal delivery” and “women without a previous vaginal delivery” were analyzed, these differences were not present. Only in women with a history of vaginal delivery and caesarean section, the rate of umbilical cord pH < 7.10 was higher (4.5% vs 1.8%, p = 0.0436). These results are partly in line with the findings in published literature. In several studies, no significant difference in Apgar score ≤ 6 or 7 after 5 min between newborns after TOLAC compared to newborns after ERCS were found [28–30]. In a retrospective cohort from Finland, the TOLAC group was compared to women with a successful vaginal delivery in history attempting a vaginal delivery again in the second pregnancy [23]. Here, pH < 7.00 was less frequently found in women with TOLAC (6.5% vs. 11.6%, p < 0.001), but perinatal mortality (0.5% vs. 0.3%, p < 0.0001), Apgar score < 6 after 5 min (3.6% vs. 1.1%, p < 0.0001) and transfer to neonatal intensive care unit (13.2% vs. 6.8%, p < 0.0001) were higher. The odds ratios for neonatal care unit treatment, perinatal mortality and neonatal mortality were 2.05 (CI 1.98–2.14), 2.15 (CI 1.79–2.57) and 1.75 (CI 1.20–2.49) [23]. In contrast to this, Kamath et al. demonstrated a higher admission rate to neonatal intensive care unit in newborns after ERCS compared to newborns born via vaginal delivery after caesarean section [31]. Overall, data concerning Apgar and umbilical blood pH after TOLAC are scarce and the definition of asphyxia inconsistent [24].

This study has its limitations and strengths. The main limitation is its retrospective design. Although the demographic parameters of the two groups differed to some extent, these differences were not clinically relevant. Comparability was therefore ensured. Remarkable strengths were the size of included cases and that standardized obstetric procedure in accordance with national guidelines was applied over the entire period. Furthermore, in contrast to most comparable studies, in the present one, there were more different maternal and perinatal outcome parameters evaluated. The results of this study are also valuable because the outcome of women with TOLAC was compared with women without a caesarean section in history achieving vaginal delivery. As other risk factors such as hypertensive pregnancy disorders and inductions of labor as well as small and large for gestational age fetuses were excluded, the previous caesarean section was the only relevant risk factor. Thus, apart from the previous caesarean section, it was a low-risk collective. The results of this study show that TOLAC is associated with a higher risk of adverse outcome. This was particularly the case for women who had not previously had a vaginal delivery. However, even in the group of women with a previous vaginal delivery and a previous caesarean section, an adverse composite outcome occurred in one in ten births. In particular, newborns had to be transferred to a neonatal care unit after birth in 7.5% and caesarean section had to be performed during the course of the birth in around 4%. The rates of meconium stained liquor and abnormal CTG were also clinically relevant (12.2% and 8.1%). The results of this study therefore show that TOLAC should only be performed in a setting where immediate pediatric care and an immediate caesarean section are possible.

## Conclusion

A previous caesarean section is associated with a higher risk of a composite adverse outcome, especially when there is no history of vaginal delivery. Relevant complications include a higher rate of uterine rupture and placental abruption. Due to the multitude of possible complications and necessary interventions, trial of labor after caesarean section should take place in an appropriate clinical setting.
